# Complementary findings on ^18^F-FDG PET/CT and ^18^F-NaF PET/CT in a patient with Erdheim-Chester disease

**DOI:** 10.1590/0100-3984.2015.0172

**Published:** 2017

**Authors:** Daniela Sabino, Romulo Hermeto Bueno do Vale, Paulo Schiavom Duarte, Marcelo Tatit Sapienza, Carlos Alberto Buchpiguel

**Affiliations:** 1 Instituto do Câncer do Estado de São Paulo (Icesp), São Paulo, SP, Brazil.; 2 Faculdade de Medicina da Universidade de São Paulo (FMUSP), São Paulo, SP, Brazil.

Dear Editor,

A 27-year-old male presented with polydipsia, polyuria, xerostomia, and mild bone pain, being diagnosed with and treated for diabetes insipidus. Thereafter, he presented with diffuse and severe bone pain, xanthomas, xanthelasmas, exophthalmia, and cholelithiasis. After a complete medical investigation, Erdheim-Chester disease (non-Langerhans cell histiocytosis) was considered the most probable clinical diagnosis. Among the imaging exams performed, he was referred for ^18^F-FDG PET/CT and ^18^F-NaF PET/CT.

The initial ^18^F-NaF PET/CT showed that ^18^F-NaF uptake was more intense in the distal femora and throughout the tibiae, as well as in the fibulae (proximal and distal), tarsi, and maxillas, than in the other bones (Figure 1A). The ^18^F-FDG PET/CT study revealed increased glycolytic metabolism in the pituitary stalk, proximal left femur, proximal fibulae, ankle, and feet, less intense uptake being observed in other areas (Figures 1B and 1C). It is of note that the 18F-FDG PET/CT was performed 9 months after the ^18^F-NaF PET/CT, showing a heterogeneous response of the lesions to the various treatment modalities the patient underwent, and that, over the course of the follow-up, he alternated between periods of clinical stability and disease progression.

**Figure 1:**
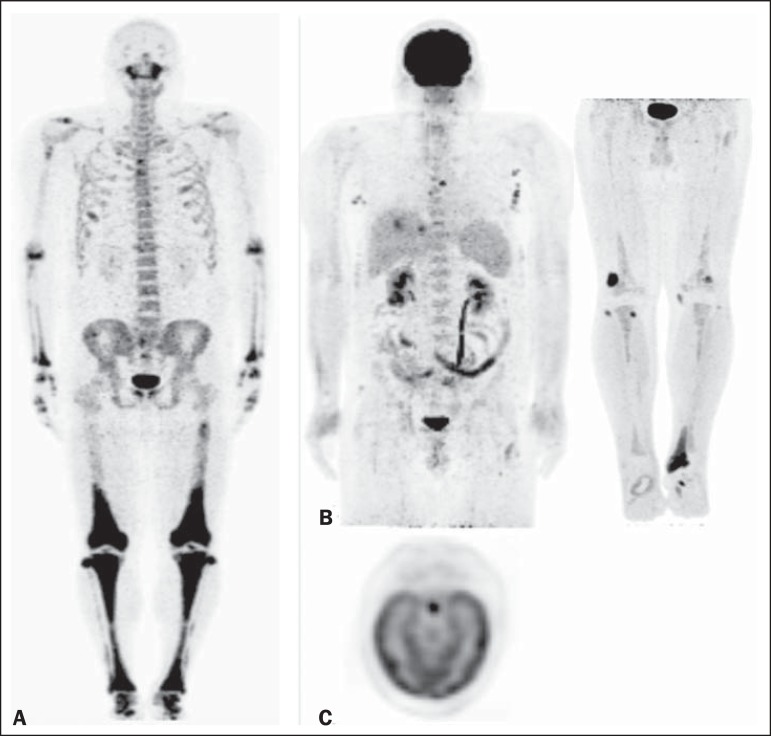
**A:** ^18^F-NaF PET/CT maximum-intensity projection image showing intense NaF uptake in the distal third of the femora; throughout the tibiae; in the proximal and distal extremities of the fibulae; in the tarsi; and in the maxillas. Note also the uptake in the proximal third of the right humerus, proximal diaphysis of the left femur, acromioclavicular joints, pubis, elbows, joints of the hands, and thoracic girdle. **B:** ^18^F-FDG PET/CT maximum-intensity projection images depicting diffuse nodular lesions in the thoracic and abdominal walls; distal metaphyseal region of the femora and tibiae; left femoral diaphysis; left Achilles tendon; and feet-the imaging criteria indicating disease progression in comparison with the findings of previous exams (not shown). **C:** PET axial brain image demonstrating high FDG uptake in the hypophysis, corresponding with the nodular thickening of the pituitary stalk seen on an MRI scan (not shown).

Erdheim-Chester disease is systemic, although variable in extent, and bone involvement is quite typical. Classical radiological findings include sclerotic and osteolytic lesions in the cortical layer of long bones, occurring bilaterally and symmetrically in their metaphysis and diaphysis, sparing the epiphysis and the axial skeleton. Approximately 50% of patients with Erdheim-Chester disease present extraosseous impairment, including changes in the hypothalamus, posterior hypophysis, eyes, retroperitoneum, skin, lungs, and heart^(1)^.

^18^F-NaF PET/CT has the advantage of being a whole-body study with high sensitivity, thereby detecting bone impairment in Erdheim-Chester disease. The use of imaging methods enables clinical suspicion for early diagnosis and patient follow-up, including therapy response assessment^(2)^. In comparison with 99mTc-MDP, ^18^F-NaF shows better pharmacokinetic characteristics, including faster blood clearance and two-fold higher uptake in bone^(3)^. Data from a number of studies, all involving small patient samples, have shown that ^18^F-NaF PET has higher sensitivity and specificity than do conventional 99mTc-based bone scans^(4-7)^. In the present study, 18F-NaF PET/CT revealed some bone lesions in the ribs and arms that were not detected by ^18^F-FDG PET/CT, indicating that the former has greater sensitivity for detecting bone lesions.

In Erdheim-Chester disease, extraosseous impairment can occur in almost every organ, which suggests that ^18^F-FDG PET/CT has potential value as a diagnostic tool. However, its main advantage is probably therapy response assessment, although that has not been well established^(8)^. This imaging modality also allows guided percutaneous biopsies (by identifying areas of high metabolic activity). Therefore, the role of 18F-FDG PET/CT in the initial diagnosis of Erdheim-Chester disease remains unclear, especially because the systemic presentation patterns of the disease are extremely variable, and it is likely to prove much more valuable for patient follow-up^(8,9)^.

## References

[1] Veyssier-Belot C, Cacoub P, Caparros-Lefebvre D (1996). Erdheim-Chester disease. Clinical and radiologic characteristics
of 59 cases. Medicine (Baltimore).

[2] Caoduro C, Ungureanu CM, Rudenko B (2013). 18F-fluoride PET/CT aspect of an unusual case of Erdheim-Chester
disease with histologic features of Langerhans cell
histiocytosis. Clin Nucl Med.

[3] Segall G, Delbeke D, Stabin MG (2010). SNM practice guideline for sodium 18F-fluoride PET/CT bone scans
1.0. J Nucl Med.

[4] Hetzel M, Arslandemir C, König HH (2003). ^18^F-NaF PET for detection of bone metastases in lung
cancer: accuracy, cost-effectiveness, and impact on patient
management. J Bone Miner Res.

[5] Hoh CK, Hawkins RA, Dahlbom M (1993). Whole body skeletal imaging with [18F]fluoride ion and
PET. J Comput Assist Tomogr.

[6] Langsteger W, Heinisch M, Fogelman I (2006). The role of fluorodeoxyglucose, 18F-dihydroxyphenylalanine,
18F-choline, and 18F-fluoride in bone imaging with emphasis on prostate and
breast. Semin Nucl Med.

[7] Schirrmeister H, Guhlmann A, Kotzerke J (1999). Early detection and accurate description of extent of metastatic
bone disease in breast cancer with fluoride ion and positron emission
tomography. J Clin Oncol.

[8] Arnaud L, Malek Z, Archambaud F (2009). 18F-fluorodeoxyglucose-positron emission tomography scanning is
more useful in followup than in the initial assessment of patients with
Erdheim-Chester disease. Arthritis Rheum.

[9] Campochiaro C, Tomelleri A, Cavalli G (2015). Erdheim-Chester disease. Eur J Intern Med.

